# Behavioral, biochemical and histopathological toxic profiles induced by sub-chronic cannabimimetic WIN55, 212–2 administration in mice

**DOI:** 10.1186/s40360-023-00644-3

**Published:** 2023-02-07

**Authors:** Ghada A. Omran, Eman S. H. Abd Allah, Sherine Ahmed Mohammed, Doaa M. El Shehaby

**Affiliations:** 1 Forensic Medicine & Clinical Toxicology Department, Faculty of Medicine, Assiut University, Assiut, Egypt; 2 Medical Physiology Department, Faculty of Medicine, Assiut University, Assiut, Egypt; 3 Medical Histology Department, Faculty of Medicine, Sohag University, Sohag, Egypt

**Keywords:** WIN55, 212–2, behavioral, Histopathologic, Biochemical, Mice, GABA/glutamate, CB1 immunohistochemistry, Gender

## Abstract

WIN55, 212–2 mesylate is a synthetic cannabinoid (SC) agonist of CB1 and CB2 receptors with much higher affinity to CB1 receptor than tetrahydrocannabinol and many potential therapeutic effects. Few studies have evaluated SCs effects on more complex animal behavior and sex differences in cannabinoids toxicology. The current study was undertaken for determination of behavioral (Open Field test), biochemical (liver and kidney function test plus GABA & Glutamate levels), histopathological and CB1 immunohistochemistry risks of sub-chronic administration of SC WIN55, 212–2 mesylate in male and female mice. A total of 40 healthy adult mice were randomly divided into four groups (5 mice each): a negative control group, a vehicle group, a low dose (0.05 mg/kg) group and a high dose group (0.1 mg/kg) for each gender.

*Open Field Test* revealed dose and gender-dependent anxiogenic effect with reduced locomotor activity in both sexes especially the higher doses with female mice being less compromised. GABA and glutamate levels increased significantly in both dose groups compared to controls alongside female mice versus males. No significant biochemical alterations were found in all groups with minimal histopathological changes. The CB1 receptors immunohistochemistry revealed a significant increase in the number of CB1 positive neurons in both low and high dose groups against controls with higher expression in female brains.

**Conclusions**

There were sexual dimorphism effects induced by sub-chronic exposure to WIN55, 212–2 with lesser female mice affection and dose-dependent influences.

**Supplementary Information:**

The online version contains supplementary material available at 10.1186/s40360-023-00644-3.

## Introduction

Synthetic cannabinoids (SCs) are psychoactive substances that are gaining high popularity due to their availability and lack of detection by standardized drug tests in addition to their potential therapeutics [1]. Although some users may realize SCs as safer substitutes to marijuana, some of them are more potent with even more serious toxicities. Many SCs have demonstrated higher affinities to the CB_1_ and CB_2_ receptors when compared to the active ingredient in cannabis, the tetrahydrocannabinol (THC) [2] Tetrahydrocannabinol displays partial agonism at the CB_1_ and CB_2_ receptors, while SCs are full agonists at these receptors [3]. Pharmacological effects of SCs are 2–100 times more potent than delta tetrahydrocannabinol (Δ9-THC), including analgesic, anti-seizure, anti-inflammatory, and anti-cancer growth effects [4].

The cannabinoid receptor (CBR) is one of the molecules related to learning and memory. They are G-protein-coupled receptor family, with two CBR subtypes: type 1 (CB_1_Ra), which is abundantly expressed in the mammalian central nervous system (CNS) [5]. The CB1 receptor is found at particularly high levels in the neocortex, hippocampus, basal ganglia, cerebellum and brainstem [6]. The type 2 is CB2 receptors which are largely restricted to immune and hematopoietic cells, the myocardium, gut, endothelial, vascular smooth muscle, Kupffer cells, bone, reproductive organs/cells, exocrine and endocrine pancreas [7]. The studies have suggested that modulating the activity of the endocannabinoid system holds therapeutic promise for a broad range of diseases, including neurodegenerative, inflammatory, and cardiovascular disorders. Cannabinoid CB1 receptors play a significant role in the modulation of Gama Amino Butyric Acid (GABA) and glutamate neurotransmission [8].

WIN55, 212–2 mesylate is a synthetic cannabinoid that is CB1 and CB2 receptors agonist with much higher affinity than Δ9-THC for CB1 receptor. It is a potent analgesic in a rat model of neuropathic pain and preventing the inflammation caused by amyloid beta proteins involved in Alzheimer’ disease. Cannabinoids can be used therapeutically in several diseases. For instance, to treat psychotic symptoms, epilepsy and spasticity as a symptom of multiple sclerosis [9]. Cannabinoids are important for patients with cancer treatment because cannabinoids have anxiogenic as well as analgesic effects so can relieve pain and reduce nausea because of chemotherapy [10]. Furthermore, they can modulate several properties of cancer cells as adhesion, invasion, migration and metastasis, as wells as tumor neovascularization [11]. Moreover, cannabinoids have the potential to influence cancer cell survival directly through inhibition of tumor cell proliferation or induction of cellular apoptosis. Beneficial effects were demonstrated in breast, skin, pancreatic, bone, oral, thyroid, prostate and lung cancers, lymphomas and gliomas after application of different cannabinoids [12, 13].

Few studies are present that evaluated SCs effects on more complex animal behavior. Still, the in vitro and in vivo studies are needed to fill in important gaps in our knowledge of tissue/organ distribution, elimination, and metabolites [14]. Furthermore, few research could emphasize characterization of sex differences in cannabinoid toxicology. Studies examining the involvement of cannabinoids in memory processes advance our understanding of the potential harmful consequences of their use and the mechanisms underlying the close relationship between cannabinoids and cognition [15, 16]. This will help in maximizing the clinical benefits of therapeutic cannabinoids, and to better handle the possible emerging deficits. The current study was undertaken to determine the behavioral, biochemical and histopathological risks of sub-chronic administration of synthetic cannabinoid WIN55, 212–2 mesylate in male and female mice. Assessment of the effects of sub-chronic administration of two dose regimens of WIN55, 212–2 on cannabinoid receptor CB1 expression, and on levels of glutamate and GABA neurotransmitters in the brain tissue in each gender were investigated.

## Materials & methods

### Animals and treatment

A total of 40 healthy adult mice (20 males + 20 females), 3–4 weeks old with average weight (25 ± 5 g) were purchased from the Faculty of Medicine’s Animal House, Assiut University. The animals were kept under a temperature of 21 ± 2 °C with a relative humidity of 50–60% and a 12 hr light/dark cycle. Adult mice were maintained under optimal laboratory conditions in two separate areas, one for each sex to avoid gender dependent odour signals and related behavioural distress. Food and water were provided for ad libitum consumption. All the experiments were performed in accordance with the protocols of the National Research Council committee regarding the international guidelines for care and use of laboratory animals in research and ARRIVE guidelines for reporting animal experiments [17, 18]. The followed protocol was also approved through the Ethical Committee of the Faculty of Medicine, Assiut University (no: 17300118).

The animals were randomly divided into four groups. Random numbers were generated using the standard = RAND () function in Microsoft Excel program as the following:

Group 1: a control group, contained 10 mice (5 males + 5 females) received nothing except a laboratory diet. Group 2: a vehicle group, contained 10 mice (5 males + 5 females) received vehicle saline + 10% dimethyl sulfoxide (DMSO) + 0.1% Tween 20. Group 3: a low dose WIN55, 212–2 treatment group that contained 10 mice (5 males + 5 females), received 0.05 mg/kg of WIN55, 212–2. Group 4: a higher dose WIN55, 212–2 treatment group contained 10 mice (5 males + 5 females) receiving 0.1 mg/kg of WIN55, 212–2. WIN55, 212–2 mesylate (Tocris, UK) was dissolved in of a vehicle solution composed of saline with 10% dimethyl sulfoxide (DMSO) and 0.1% Tween 20 (Sigma Aldrich, USA). WIN55, 212–2 solution was injected daily intraperitoneal (I.P) in an injection volume of 0.05 ml/kg and 0.1 ml/kg. The doses of both compounds were chosen to avoid side effects like catalepsy, motor impairment, or sedation [19]. Male mice groups were processed separately followed by female mice groups in all downstream analyses.

The experiment lasted 28 days, by the end of which (last day), a neurobehavioral test (OFT) was performed 30 min after last injection, then by the end of the 24 h of the same day, blood samples were withdrawn for biochemical analyses then organs dissection were undertaken after animals’ euthanasia. To achieve a blinded assessment, each animal was evaluated by four different investigators as follows: a first investigator administered the treatments based on the randomization table. This investigator was the only person aware of the treatment groups allocation. A second investigator was responsible for the behavioural test procedure, whereas a third investigator performed the histopathological and immunohistochemistry procedures. Finally, a fourth investigator (also unaware of treatment) collected the open field test results, the GABA/glutamate levels, liver & kidney functions, organs histopathology and brain CB1 immunohistochemistry results*.* All mice were subjected to the following assessments.

### Behavioural assessment

The Open Field Test (OFT) is a commonly used test to assess anxiety-like behavior, locomotion, and exploration behavior. The apparatus is a wooden, open-topped box (72 cm × 72 cm × 37 cm). The open field arena is divided into equal sized squares (4 X 4) & a central square is drawn in the center. Before the experiment, each mouse was placed separately in an empty square for 5 min for acclimatization. During the test, each mouse was placed in the center of the apparatus and a video was recorded which was later scored for the number and duration of central square entries and the number of lines crossed in 10 min. Between each examination, 70% ethyl alcohol was used for cleaning the apparatus. Decreased number of squares crossed indicate decreased locomotor activity. Decreased central square entries and duration indicates increased level of anxiety [20, 21].

### Biochemical assessment

Blood samples were taken from the retro-orbital sinus under light phenobarbital anesthesia, centrifuged at 3000 rounds per minute (rpm) for 15 min and the clear, non-hemolyzed supernatant sera were removed and kept at − 20 °C until use. Then, euthanasia was undertaken by cervical dislocation. Kidney and liver functions of all mice of the two sexes in different dose groups were determined using colorimetric kits for serum urea, creatinine, AST and ALT (Biodianostics, Cairo, Egypt) and a suitable spectrophotometer (Pharmacia LBK Ultrospec III, Biochrom, Cambourne, England).

### Histopathological examination

Mice brains were quickly dissected and transferred to a wetted filter paper and hemisected into two sagittal halves. The first part was washed with ice-cold isotonic saline solution 0.9%, frozen in liquid nitrogen, and kept at − 80 °C for subsequent use in biochemical assessment of glutamate and GABA in the prefrontal cortex, basolateral amygdala and ventral hippocampus; that were dissected according to previously described methods [22, 23]. These brain regions were known to belong to the emotional circuit and contain high levels of CB1 receptors. The other brain halves were rapidly processed and kept in phosphate buffer (PH 7.4) containing 4% formaldehyde [24] for use in histopathological examination and immunohistochemical staining for CB1 expression assessment specially in the emotional circuit of aforementioned regions.

Livers were excised and a 1 mm section was obtained from the right lobe. Kidneys were excised 1 mm section from the left and right kidneys as well. The specimens from each animal were fixed in 10% formalin solution for 24 h, then briefly rinsed in PBS and stored in 70% ethanol for 24 h before specimens were processed for light microscopic study. Paraffin sections (5 μm thick) were stained with the following and evaluated in a blinded fashion:

1. Hematoxylin and Eosin staining of the brain, liver and kidney of both mice sexes [25].
2. Immunohistochemical staining CB1 receptor antibody (1:1000) for brains: deparaffinized and rehydrated sections were blocked in hydrogen peroxide 3% then incubated for 20 min in citrate buffer (pH 6.0) for antigen retrieval. Slides were incubated overnight at 4 °C in a humid chamber with the polyclonal primary anti-cannabinoid receptor 1/CR1 antibody (Booster Bio, USA, A01291–1). The modified Avidin–Biotin immune-peroxidase technique was used. Sections were counterstained with Mayer’s hematoxylin, then dehydrated, cleared, and mounted. Negative control experiments were performed by omitting the primary antibody. The positive results were indicated by brown coloration [26]. The light microscope Leica ICC50 Wetzlar (Germany) was recruited, with ten high power fields (× 400) for each section in all groups were evaluated. Analysis of each field was undertaken using Image J 1.51n software (National institute of health, USA, Java 1.8.0_66, 32-bit) for counting positive cells.

### HPLC analysis of GABA& glutamate in mice brain

The dissected brain regions involved in emotional circuits were separated, weighed, and placed in 1.5 ml microcentrifuge tubes for HPLC analysis and fluorescence detection. The samples were homogenized in 15 volumes of methanol/water mixture (85:15, v/v); then centrifuged at 7800×g for 15 min at 4 °C, and aliquots of the supernatants were stored at − 20 °C until GABA/glutamate analysis. Appropriate GABA and glutamate standards were preprepared [27]. Derivatization was performed by mixing 100 μL sample or standards solutions, 20 μL of prepared methanolic phthalaldehyde (OPA) in concentration of 5 mg/mL, 75 μL borate buffer (pH 9.9) and 5 μL of 3-mercaptopropionic acid (MPA). The resulting solution was vortexed and analyzed after 1 min at room temperature before high performance liquid chromatography analysis [28]. The used HPLC system consists of an Agilent Technologies 1200 series system (Waldbronn, Germany) encompassing a zobrax extended C18 analytical column (150 mm × 4.6 mm) and a fluorescence detector (G1321 A; Waldbronn, Germany). The mobile phase consisted of a mixture of 0.05 M sodium acetate, tetrahydrofuran and methanol (50:1:49, v/v) adjusted to pH 4.0. The mixture was filtered through 0.45 μm Durapore membrane filters (Millipore, USA). Compounds were eluted isocratically over a 9 min runtime at a flow rate of 1 mL/min. The fluorescent detector was set at an excitation wavelength of 337 nm and an emission wavelength of 454 nm. Obtained values are reported in μg/g of brain tissue [27]. All chemicals and solvents used were purchased from Sigma-Aldrich, USA.

### Statistical analysis

Statistical analysis of data was performed by SPSS ver. 19 (Chicago, USA) and Graph Pad Prism 5 Software (San Diego, California, USA). All data were expressed as mean values ± standard deviation. Before the assessment, the variables were checked for normal distribution with the Shapiro-Wilk W test. Changes between samples were compared either by Student’s t-test in analyzing gender in relation to brain amino acids (glutamate & GABA) levels with each of the two doses of WIN55, 212–2; or determined by two-way ANOVA (analysis of variance) for main effects of gender, treatment (none, vehicle (saline + DMSO) or (low or high doses), and their interaction for behavioral investigation. Otherwise, one-way ANOVA was carried out. Post-hoc test (Least Significant Difference; LSD) was applied for multiple comparisons among the studied groups. *P* value < 0.05 was considered statistically significant.

## Results

### Behavioral assessment

**Fig. 1:**
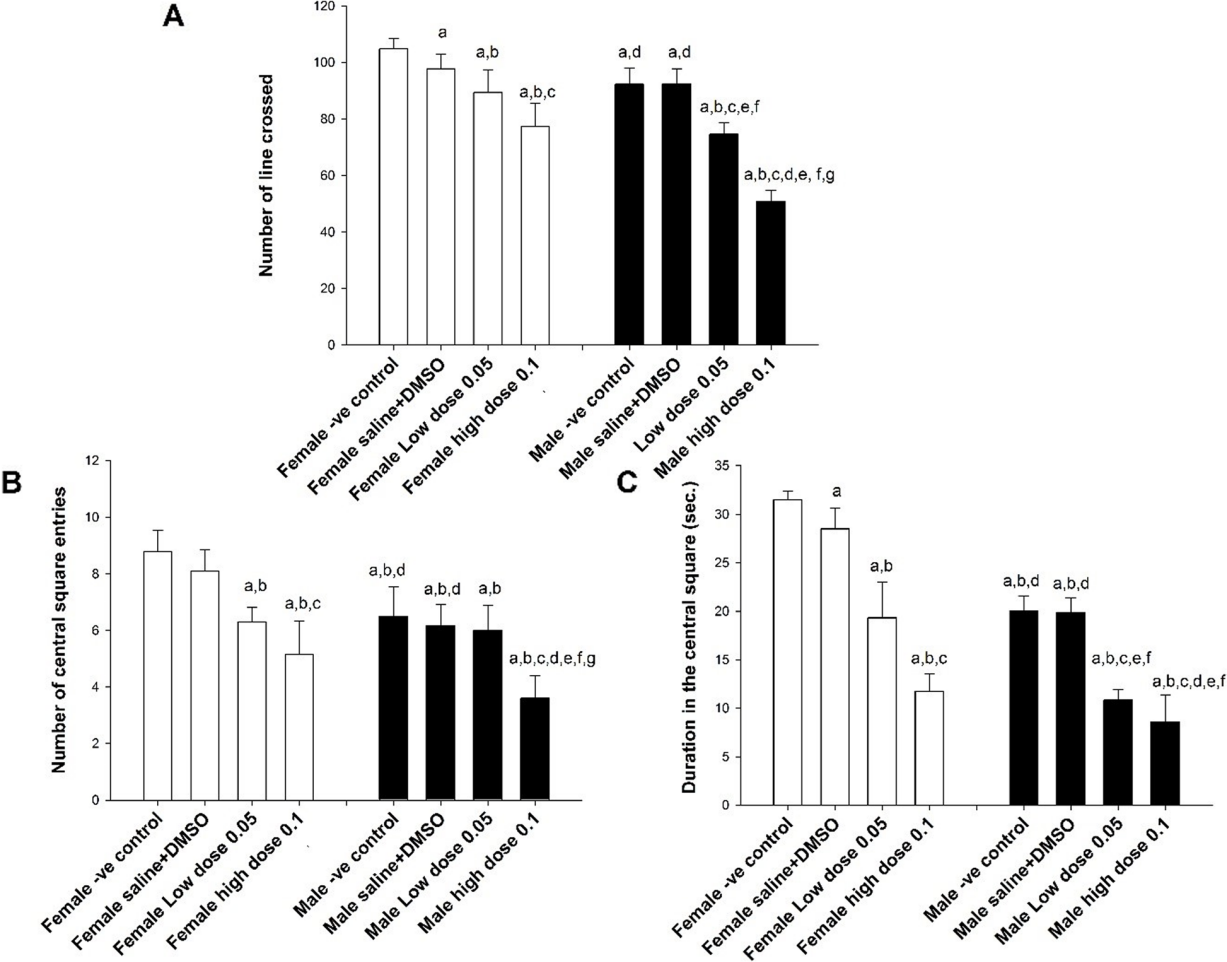
Open Field Test results of WIN55, 212–2 treated mice versus controls. The number of lines crossed (**A**), the number of central square entries (**B**), and the duration in the central square (**C**) in the groups studied are displayed. Mean ± SD are shown. Statistical differences were determined by two-way ANOVA (analysis of variance). Post-hoc test (Least Significant Difference; LSD) was carried out to determine differences between groups. ^a^Statistically significant as compared to the female -ve controls. ^b^Statistically significant as compared to the female vehicle group (saline and DMSO). ^c^Statistically significant as compared to the female low dose group (0.05 mg/kg). ^d^Statistically significant as compared to the female high dose group (0.1 mg/kg). ^e^Statistically significant as compared to the male -ve controls. ^f^Statistically significant as compared to the male vehicle group (saline and DMSO). ^g^Statistically significant as compared to the male low dose  group (0.05mg/kg).

Two-way ANOVA results of the *Open Field Test* revealed a significant effect of gender on the locomotor activity indicated by the number of lines crossed (F (1,40) =78.5, *p* < 0.001), and a significant effect of treatment as well (F (3,40) =86.9, *p* < 0.001) with a confirmed interaction (F (3,36) =6.7, *p* = 0.001) (Fig. 1). Female mice had significantly higher locomotor activity compared to males (*p* < 0.001). Moreover, administration of WIN55, 212–2, either in low or high dose, significantly reduced the locomotor activity compared to controls (*p* = 0.001); however, the higher dose of WIN55, 212–2, was associated significantly wither further reduction of the locomotor activity compared to the low dose (*p* < 0.001) (Fig. 1A).

There was a significant effect of gender (F (1,40) =38.67, *p* < 0.001) and treatment (F (3,40) =33.47, *p* < 0.001) on the number of central square entries, with a significant interaction (F (3,40) =3.11, *p* < 0.05). Generally, female mice had a higher number of central square entries (less anxious) compared to male mice (*p* < 0.001). Mice administrated WIN55, 212–2, either the low or high dose, had a lower number of central square entries (i.e. more anxious) compared to the controls (*p* < 0.001). The higher dose of WIN55, 212–2 resulted in a significantly lower number of central entries compared to the low dose (*p* < 0.001) (Fig. 1B).

Regarding the duration spent in the central square, there were significant effects of gender (F (1,40) =164.5, *p* < 0.001) and treatment (F (3,40) =145.82, *p* < 0.001), with a significant interaction (F (3,40) =7.99, *p* < 0.05). Female mice spent more time in the central square compared with male mice (*p* < 0.001). Mice receiving WIN55, 212–2, either low or high dose, spent less time in the central square than control mice (*p* < 0.001); however, the effect of the high dose was more prominent (*p* < 0.001) (Fig. 1C).

### Biochemical assessment

#### Proteomic HPLC analysis of glutamate and GABA in the brain

In Table 1, WIN55, 212–2 treated male mice at low and high doses showed a statistically significant increase in glutamate level in comparison with control and vehicle groups (*p* < 0.0001) for all group comparisons. Concerning GABA levels between groups; a significant stepwise increase was found between all groups (*p* < 0.001& < 0.001 for control & vehicle groups respectively; *p* < 0.0001& < 0.0001 for WIN55, 212–2 treated male mice at the two dose groups, respectively). On the other hand, WIN55, 212–2 treated female mice showed non-significant difference of glutamate and GABA levels between the control and vehicle groups, while low and high doses groups of WIN55, 212–2 treated female mice showed a significant increase of glutamate and GABA levels in comparison with control and vehicle groups (*p* < 0.0001). Table 2 showed significant higher levels of glutamate and GABA neurotransmitters in female mice treated with low and high doses of WIN55, 212–2 (*p* < 0.0001& *p* < 0.0001 respectively).

**Table 1:** Proteomic analysis of brains’ glutamate and GABA levels of WIN55, 212–2 treated mice

Gender	Control Mean ± SD	Vehicle Mean ± SD	WIN55, 212–2 (0.05 mg/kg) Mean ± SD	WIN55, 212–2 (0.1 mg/kg) Mean ± SD
**Male mice**				
Glutamate	0.072 ± 0.013 ^b**c***d***^	0.095 ± 0.005^a**,c***,d***^	0.126 ± 0.022^a*** b*** d***^	0.272 ± 0.010 ^a***b***c***^
GABA	0.430 ± 0.003 ^b***c***d***^	1.60 ± 0.001^a***,c***,d***^	7.40 ± 0.230^a***b*** d***^	10.1 ± 0.79 ^a***b***c***^
**Female mice**				
Glutamate	0.59 ± 0.097^bNS c***d***^	0.56 ± 0.097 ^a NS c*** d***^	5.41 ± 4.65 ^a** b*** d***^	11.59 ± 4.11^a*** b*** c***^
GABA	9.25 ± 0.594^bNS c***d***^	9.86 ± 0.878^aNS,c***, d***^	12.7 ± 1.17 ^a***.b***,d***^	23.8 ± 2.21 ^a***,b***,c***^

One-way ANOVA test was undertaken for each mice sex. Data expressed as mean ± SD

NS: non-significant

^*^p < 0.05

^**^p < 0.01

^***^p < 0.001

^a^comparison with the control group

^b^comparison with the vehicle group

^c^comparison with the low dose group

^d^comparison with high dose group

**Table 2:** Comparison of glutamate & GABA levels of male and female treated mice with WIN55, 212–2 in two doses regimen

	WIN55, 212–2 0.05 mg/k (Low dose)		*p-value*	WIN55, 212–2 0.1 mg/kg (High dose)		*p*-*value*
Group	Male	Female		Male	Female	
Glutamate	0.126 ± 0.022	5.41 ± 4.6	< 0.0001	0.2717 ± 0.01	11.59 ± 4.11	< 0.0001
GABA	7.40 ± 0.23	12.7 ± 1.17		10.1 ± 0.79	23.8 ± 2.21	

Independent sample student t- test was carried out, values represent means + SD

#### Liver and kidney function tests

Biochemical analysis of hepatic enzymes in the serum revealed non-significant increase in AST and ALT in WIN55, 212–2 treated mice compared to the control and vehicle groups. However, this increment was more observed in high dose groups than the low dose groups. Biochemical analysis of hepatic enzymes in the serum revealed, a non-significant increase in AST and ALT in WIN55, 212–2 treated mice of both sexes compared to the control and vehicle groups, this increase is more in high dose than the low dose groups. Biochemical analysis of blood urea and serum creatinine revealed non-significant increase in WIN55, 212–2 treated mice compared to the control and vehicle groups, which was also more in high dose groups (Table 3).

**Table 3:** Biochemical assessment of liver and kidney functions in WIN55, 212–2 treated mice versus controls

Test	Control	Vehicle	WIN55, 212–2 0.05 mg/kg	WIN55, 212–2 0.1 mg/kg	*P*-value
ALT	17.1 ± 1.92	17.9 ± 1.93	18.8 ± 2.56	19.8 ± 2.56	NS
AST	52.8 ± 2.43	56.8 ± 2.06	57.4 ± 2.31	59.2 ± 0.82	NS
Blood urea	40.60 ± 15.23	44.6 ± 18.45	45.10 ± 14.82	47.60 ± 10.73	NS
Serum creatinine	0.13 ± 0.03	0.14 ± 0.06	0.16 ± 0.04	0.18 ± 0.05	NS

One-way ANOVA test, data are expressed as mean ± SD

NS: non-significant

### Histopathological examination

**Fig. 2:**
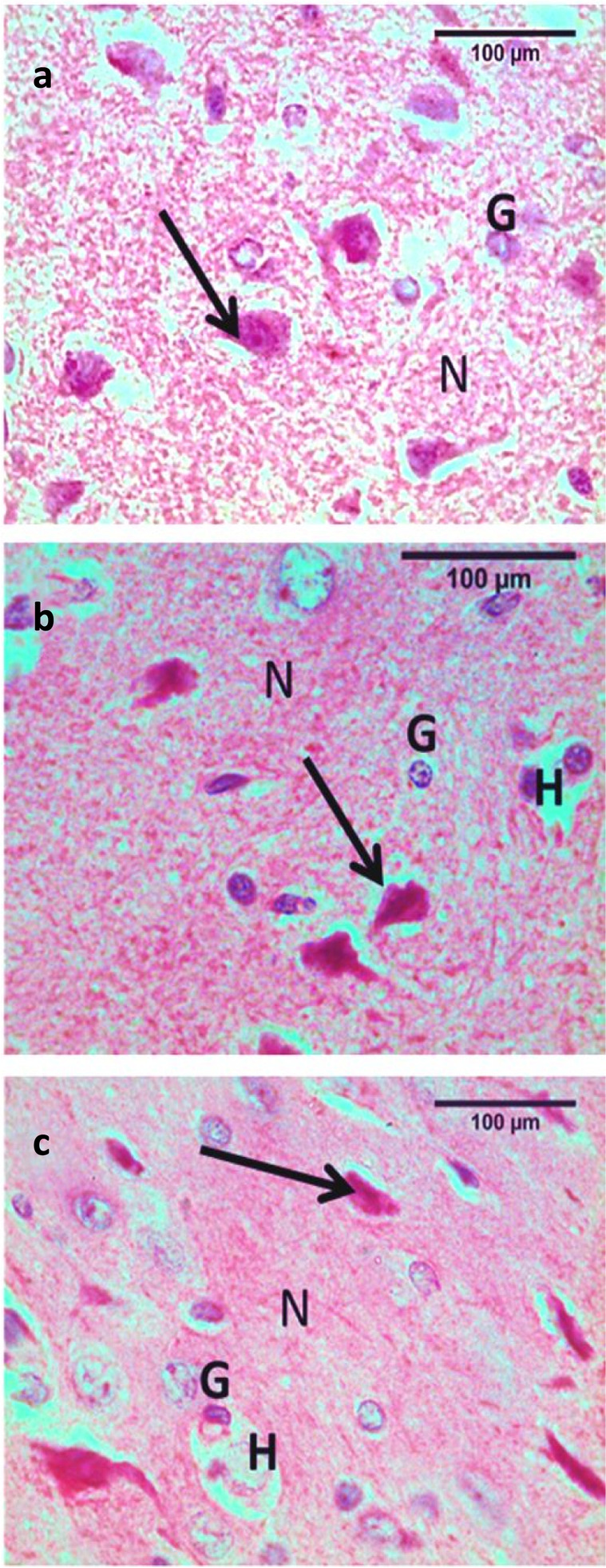
Photomicrographs of cortical brain sections of WIN55, 212–2 treated mice versus controls. Image a: control and vehicle groups showing rounded non pyramidal granule cells (G) with open face nuclei and large sized pyramidal cells (arrow) with triangular shaped cell bodies, basophilic cytoplasm, rounded nuclei, and long apical dendrites. The ground substance between the nerve cells was normally occupied with homogenous eosinophilic neuropil (N). Image b: the low dose groups showing some pyramidal cells are deformed with deeply stained nuclei and surrounded by haloes (arrow), granule cells (G) with dark smaller nuclei surrounded by halo (H) nuclei and neuropil (N) in between cells. Image c: the high dose groups show more deformed and shrinked pyramidal cells (arrow), granule cells (G) with dark smaller nuclei surrounded by halo (H) nuclei and neuropil (N) in between cells. Stain H&E (scale bar = 100um)

The brain cortex in the control and vehicle groups contained neurons, glial cells and nerve fibers. The organization of neurons in the cerebral cortex appeared as six layers; the molecular layer (fibrous with few nerve cell bodies), the external granular layer, the external pyramidal cell layer, the internal granular layer, the internal pyramidal cell layer and the multiform cell layer. The neuropil contained neuroglia, nerve fibers and blood vessels with a narrow perivascular space. Cortical neurons had rounded vesicular nuclei with prominent nucleoli, and rounded granule cells. The pyramidal cells were pyramidal in shape with a slight basophilic cytoplasm and a large apical dendrite (Fig. 2a). In the low dose groups, some nerve cells showed degeneration. Degenerated pyramidal cells were deformed with deeply stained nuclei, surrounded by haloes. Degenerated granule cells appeared with dark smaller nuclei surrounded by haloes (Fig. 2b). In the high dose groups, more degenerated cells were seen (Fig. 2c).

**Fig. 3:**
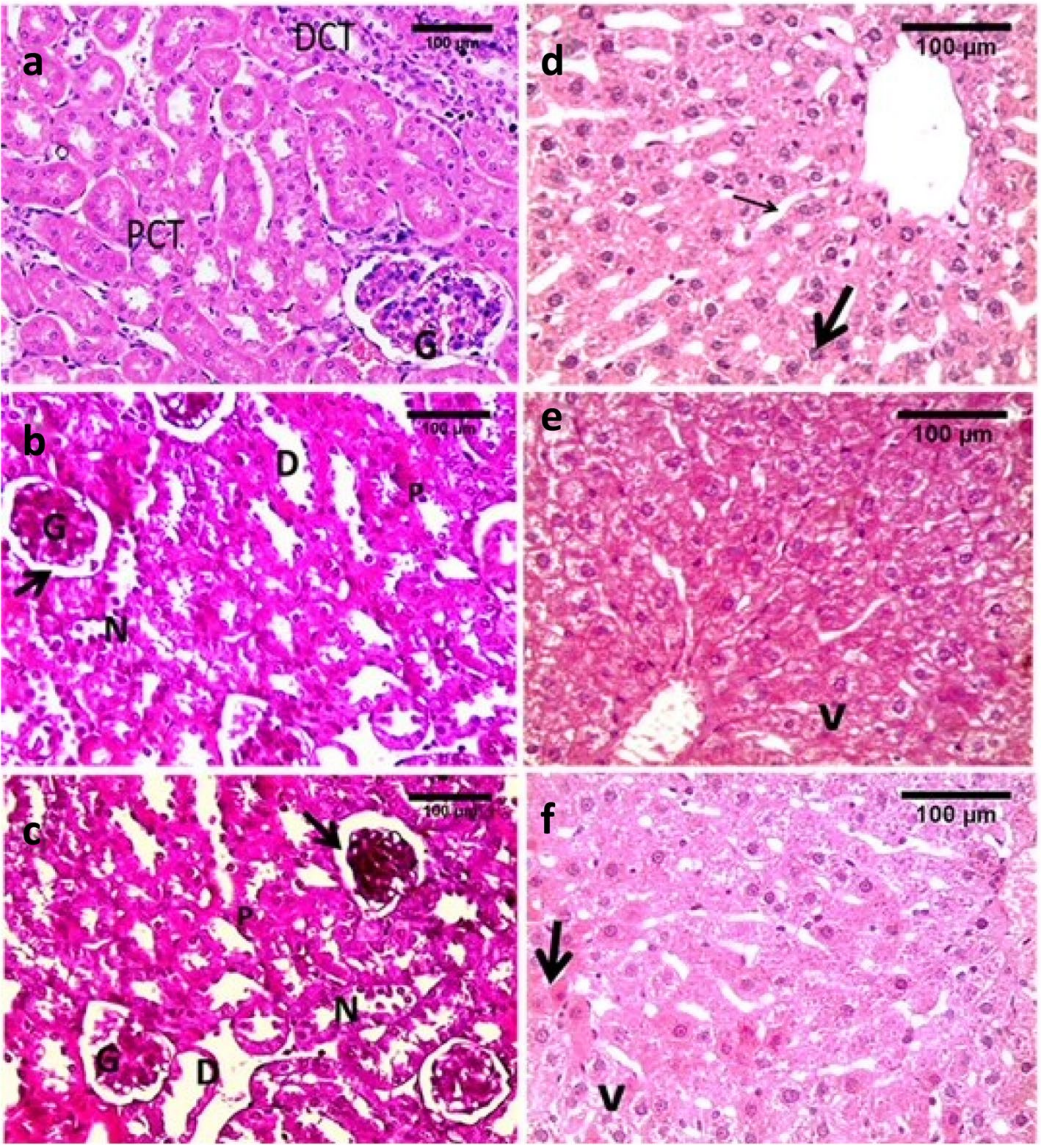
Photomicrographs of liver and kidney sections of WIN55, 212–2 treated mice and controls. Control and vehicle groups: **a** & **d,** low dose groups: **b** & **e** and high dose groups: **c**& **f.** Image **a** shows normal kidney, proximal convoluted tubule (PCT), distal convoluted tubules (DCT) and glomeruli (G). Image **b** low dose groups showing some degenerative histological changes in the kidney compared to controls. Image **c** changes are more pronounced in the high dose groups with shrinkage of glomeruli (G). Changes in **b** &**c** were in the form of tubular epithelium degeneration and necrosis (N), tubular dilatation (D) with dilatation of urinary space in Bowman’s capsule (arrow) and pyknotic nuclei (p). Image **d** shows normal hepatocytes with acidophilic cytoplasm (thick arrow) and blood sinusoids in between hepatocytes (thin arrow). Image **e** reveals vacuolated cytoplasm of hepatocytes (v) in low dose groups. Image **f** display vacuolated hepatocytes (v) in high dose and some nuclei are pyknotic (arrow). Stain H&E (scale bar = 100 um)

In the kidney sections, the control and vehicle groups showed normal renal corpuscles with the proximal and distal convoluted tubules having normal epithelium. The proximal convoluted tubules had a narrower diameter and a smaller number of epithelial cells than the distal convoluted tubules (Fig. 3a). The low dose showed minimal histopathological changes in the kidney compared to the controls in the form of tubular epithelium degeneration, necrosis, and tubular dilatation. Dilatation of the urinary space in Bowman’s capsule appeared in some renal corpuscles (Fig. 3c). These changes were more pronounced in the high WIN55, 212–2 dose with shrinkage of glomeruli and more renal corpuscles were affected (Fig. 3e).

In the liver sections, the control and vehicle groups showed normal histological structure with normal architecture hepatocytes. The latter showed central rounded vesicular nuclei, slightly vacuolated acidophilic cytoplasm, and were radiating from the central vein. The portal triads were seen at the periphery of the hepatic lobule (Fig. 3b). Minimal changes in the form of some degenerated hepatocytes with vacuolated cytoplasm were seen in low doses with some disturbed architecture of the liver (Fig. 3d). Hepatocyte degeneration was more pronounced in the high WIN55, 212–2 dose than in the low dose groups. More vacuolated hepatocytes and more disturbance of architecture were seen in the high dose group. Cells with pyknotic nuclei were seen abundantly in the high dose groups (Fig. 3f).

### Immunohistochemical studies

**Fig. 4:**
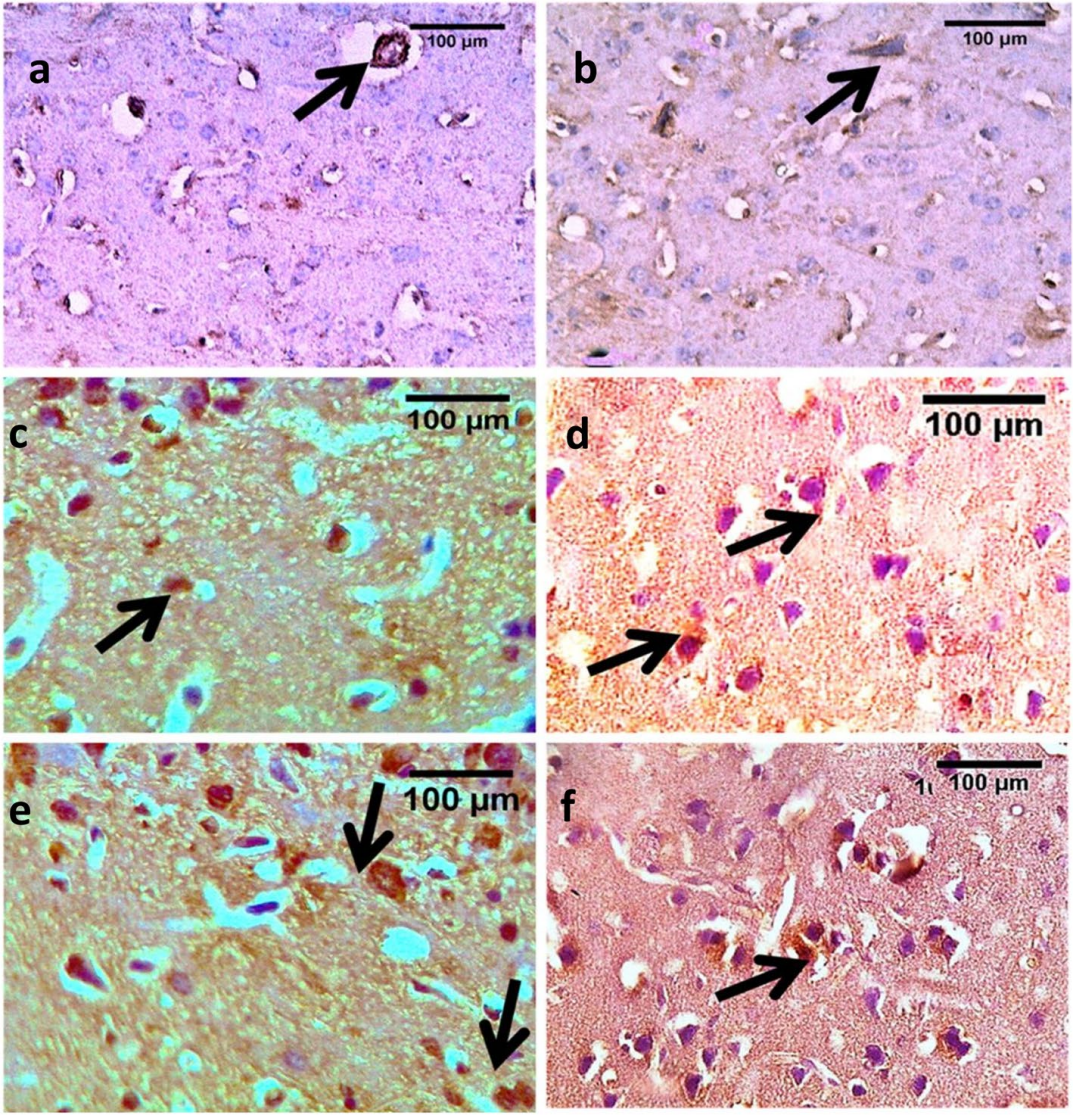
Photomicrographs of cortical brain sections for CB1 receptor immune expression in WIN55, 212–2 treated mice and controls. Images **a, c** & **e** represent female mice sections and **b, d** &**f** images represent male sections. Image **a** control and vehicle females and **b**: control and vehicle males. Controls showed more expression of CB1 receptors in female mice. Positive neurons are marked with arrows. Images **c** & **d** showed the low dose groups with higher immune expression compared to the control and vehicle groups which are more marked in female mice. Images **e** & **f**: high dose groups showed more CB1 expression than the low dose groups (scale bar = 100um)

There was significant increase in the number of CB1-positive neurons in both low and high dose groups compared to the control and vehicle groups in both sexes. Moreover, expression was significantly higher in the high WIN55, 212–2 dose groups compared to the low doses. Sex differences were observed as well in the form of significant higher CB1 receptor expression in female versus male groups (Fig. 4, Table S1).

## Discussion

The present study demonstrates gender and dose-dependent effects of the synthetic cannabinoid WIN55, 212–2 on locomotor activity, anxiety, associated cannabinoid receptor CB1 expression and neurotransmitters glutamate/GABA levels in the hippocampus and prefrontal cortex of treated mice. The current work revealed the involvement of CB1 receptor in the control of locomotor activity and anxiety as previously confirmed by Iverson and Maccarrone through the huge presence of the CB1 receptors and endocannabinoids in regions that are important for anxiety and emotion, including the amygdala and hippocampus [29, 30]. Korem and colleagues stated that the most common self-reported reason for using cannabis is rooted in its ability to reduce feelings of tension, stress, and anxiety through the endocannabinoid system and this represents an attractive approach to the treatment of anxiety-related disorders, particularly post-traumatic stress disorders [31].

The observed anxiogenic effect and reduced locomotor activity of WIN55, 212–2, CB1 agonist, were significantly dose dependent (more in the high dose than the low dose), this result is in accordance with a previously documented study that WIN55, 212–2 in 1 mg/kg, I.V significantly reduced locomotor activity [32]. Another SC agonist 5F-AMB, produced an anxiolytic effect and reduced locomotion in the Open Frame test in partial agreement with what was found here [33]. Furthermore, these effects were previously confirmed by use of CB1 receptor antagonist Rimonabant, which stimulated locomotor activity in mice [34], along with another study that revealed the genetic disruption of CB1 receptors consistently increased anxiety [35]. However, many previous animal studies showed controversy regarding cannabinoids effects on anxiety outcomes. With respect to CBD (Cannabidiol), both Schier et al. [36] and Blessing et al. [37] concluded that when it was administered acutely, anxiolytic-like effects were only present at low doses. Schier et al. [36] also noted that chronic doses produced mixed results, with both anxiolytic-like and anxiogenic-like outcomes. These variant outcomes of cannabimimetic compounds may be attributed to different study designs, the size of the apparatus used, types of tests employed, different used dosages, and routes of administration.

The anxiogenic effect and reduced locomotor activity of WIN55, 212–2, (CB1 agonist) were sex dependent being significantly less in females than males. Gender-driven anxiogenic effects were also evident in Kasten et al. in their 2019 study. They explored the anxiogenic/anxiolytic behavior of adolescent male and female mice upon acute and chronic intraperitoneal administration of different doses of THC and CBD separately or combinations. They utilized open field and elevated pulse maze tests. All tested doses (1-10 mg/kg THC & 5–20 mg/kg CBD) produced predominantly anxiogenic effects apart from CBD when used alone which was marked in female mice contrary to what was observed in current work [38]. Sex-dependent differences were frequently observed in the biological and behavioral profiles in the examined species. Castelli & coworkers in 2014 provided biochemical evidence for sex and hormone- dependent differences in the density and function of CB1 receptors in selected brain regions, revealing a more vulnerable behavioral phenotype in female than in male rats. Estradiol was identified as the hormone that contributes most to the sexually dimorphic effects of cannabinoids in adults [39]. Sex differences observed in the cannabinoid-induced effects related to cannabis abuse and dependence addressed that many, but not all sexually dimorphic effects of exogenous cannabinoids can be attributed to a sexually dimorphic endocannabinoid system in rodents [40, 41]. Several studies have also reported that cannabinoid agonists are more potent in female than male adult rats in decreasing locomotor activity [42], as well as in producing catalepsy [16, 40].

The assessed significant increase of glutamate and GABA levels in the hippocampus and prefrontal cortex in WIN55, 212–2 treated mice were dose dependent (more in the high dose than the low dose), and sex dependent (significantly more in females than males). These results were correlated with the result of many previous reports [43–47]. This could be explained by the controversial modulating action of cannabinoids on brain neurotransmitters as reviewed by Vasaghi et al. [47]. It has been shown that WIN55, 212–2 could increase the secretion of dopamine, which would also lead to the increase in the level of GABA and glutamate as well. Pollak and colleagues [45] through their study using both electrophysiological and optogenetical techniques demonstrated a “hyperdirect” excitatory pathway providing a direct excitatory control of 5-HT (serotonergic neurons), with general increase in the activities of neurotransmitters; dopamine, GABA and glutamate and this increase could have resulted from elevated brain neural activities most likely important for proper function of the 5-HT system. On the contrary to the present findings, independent studies have shown that D9-THC and other cannabinoid agonists, such as WIN55, 212–2, could induce a decrease in glutamatergic neurotransmission in the hippocampus, striatum and nucleus accumbens (NAc) [48, 49]. Marco et al. [50] confirmed the previous results through their finding in mice with a deletion of the CNR1gene, which codes for CB1 receptor proteins, show decreased GABA-A receptor levels and a reduced functionality of GABA-B receptors [50]. The sex-based marked increments of GABA and glutamate neurotransmitters in female hippocampus compared to males in the two dose regimens, were in line with a previous study that investigated adolescent cannabinoids exposure on glutamate/GABA balance in the rat’s hippocampus. Adolescent cannabinoid exposure was associated with enhanced GABA release and GABAB receptors up-regulation which result in disrupted glutamate/GABA balance in the hippocampus of female rats [51]. Further, the biphasic effect of cannabinoids on anxiety can be explained through controlling glutamate/GABA balance as they can stimulate terminals of both opposing neurotransmitters and the produced action is the outcome of their balance [52].

Sub-chronic WIN55, 212–2 administration was associated with significant immunohistochemical changes. The latter was in the form of the significant increased expression of CB1 positive neurons in both low and high dose groups that correlates with differential anxiogenic effects, reduced locomotion and the increased GABA and glutamate levels compared to control and vehicle groups, which also was more in the higher than the lower dose groups. Sex difference was observed in CB1 receptor expression with significant higher expression in female than male groups. Previous studies had reported sex and hormone-dependent differences in the density and function of CB1 receptors. Females were inherently more sensitive than males to drugs of abuse and were more susceptible to drug addiction [39].

Concerning the toxicological effects of sub-chronic WIN55, 212–2 administration in mice organs, the prefrontal cortex showed more signs of degeneration in nerve cells in the WIN55, 212–2 high dose than the low dose treated mice of both sexes. Halos of empty spaces surrounding neuronal cells in treated groups could be explained by shrinkage, necrosis and apoptosis of cells leaving pericellular spaces. The latter spaces are formed secondary to cytoskeletal affection with subsequent shrinkage of cells and withdrawal of their processes [53].

There were limited histological degenerative changes in the livers of cannabinoid-treated mice which were more pronounced in the high dose groups in each mice sex. In previous studies, 90-day oral treatment with cannabinoids (30–300 mg/kg) had little effect on rhesus monkeys’ liver and kidney without morphologic changes [54], this result is confirmed by the non-significant increase in AST and ALT levels.

In a previous study, the cannabidiol (CBD) in combination with other drugs was found to induce hepatotoxicity as diffuse sinusoidal dilation accompanied by vascular congestion, hepatic plate atrophy, and centrilobular necrosis in mice livers received CBD plus acetaminophen. However, foci of sinusoidal destruction were observed in the livers of only two out of eight mice in higher dose of that drug combination and one out of seven mice in the vehicle plus acetaminophen group. This was explained by modulation of various cytochrome P450 (CYP) and UDP-glucuronosyltransferase (UGT) enzymes responsible for xenobiotic metabolism [55]. Furthermore, dose dependent hepatocellular degeneration in mice gavaged with CBD ranged from focal cellular degenerative changes with high doses, while no cellular changes with lower doses (61.5 mg/kg cannabinoids) [55]. A comprehensive review on human studies of synthetic cannabinoids abusers revealed that SCs use can produce a non-negligible hepatotoxicity [56]. In contrast to current results, Magen and coworkers reported that cannabinoids improved toxicity in a model of chronic liver disease in a dose of 5 mg/kg intraperitoneal cannabidiol daily for 4 weeks [57]. They explained that by anti-inflammatory effect of cannabidiol and the diseased liver might have a different response to cannabinoids than the normal liver [58] .

Histopathological examination of the kidneys of treated animals revealed some degenerative changes in the form of cellular degeneration, which was more pronounced in the high dose than lower dose groups though not associated with significant biochemical changes. In a previous study, animals treated with 5 mg/kg & 10 mg/kg cannabis showed many lesions like atrophy of proximal convoluted tubules, coagulative necrosis and dilation of Bowman’s capsules, and others showed atrophy of glomeruli specially with the higher dose [59]. However, another study showed damaged pyknotic cells, degenerated distal and proximal convoluted tubule as well as disarranged glomerulus and narrowed Bowman’s capsule with 700 mg/kg cannabis treated rat groups respectively [60]. This could be justified that the kidney and liver are important organs of metabolism and detoxification of xenobiotics and their metabolites, so they are especially susceptible to damage. In contrast, a previous study reported no kidney toxicity in abusers of cannabis [61].

## Conclusions

To conclude, the evaluation of potential toxic effects on mice sexes treated with WIN55, 212–2 in the two suggested dose regimen revealed sexual dimorphism in behavior being less anxiogenic with limited reduction of locomotor/exploratory activity in females, alongside concomitant increment in neurotransmitters levels and brain CB1 receptors expression compared to male mice. This was also more pronounced in the higher dose groups. Meanwhile, minimal histopathological changes and insignificant biochemical parameters compromise in both sexes justifies the relative safety and potential beneficial therapeutic effects of suggested doses, especially the lower doses for females.

## Supplementary Information

**Additional file 1: Table S1.** Mean number of cells positive for CB1 receptor per high power field expression of brain areas involved in emotional circuit.

## Data Availability

All data analyzed through this study are included in this published article.

## Abbreviations

Δ9-THC: Δ9-tetrahydrocannabinol
SCs: Synthetic cannabinoids
CB1: Cannabinoid receptor 1
CB2: Cannabinoid receptor 2
CBR: Cannabinoid receptors
NMDA: N-methyl-d-aspartate
PBS: Phosphate buffer saline
WIN-55212–2: (R)-(+)-[2,3-dihydro-5-methyl-3-(4-morpholinylmethyl)-pyrrolo[1,2,3-de]-1,4benzoxazin-6-yl]-1-naphthalenyl-methanone mesylate
GABA: Gamma-aminobutyric acid
DMSO: Dimethyl sulphoxide
CBD: Cannabidiol
NAc: Nucleus accumbens
5-HT: 5-hydroxytryptamine (serotonin)
ALT: Alanine aminotransferase
AST: SGOT (serum glutamic-oxaloacetic transaminase)
OPA: Ortho-phthalaldehyde
MPA: 3- Mercaptopropionic acid
HPLC: High performance liquid chromatography
ANOVA: Analysis of molecular variance
LSD: Least significant difference
CNS: Central nervous system
