# Supplementary material for: Behavioral, biochemical and histopathological toxic profiles induced by sub-chronic cannabimimetic WIN55, 212–2 administration in mice
Source: BMC Pharmacol Toxicol. 2023 Feb 7;24:8. doi: 10.1186/s40360-023-00644-3 (PMC9906926; doi:10.1186/s40360-023-00644-3)
Supplement: Supplementary file 1 — Additional file 1: Table S1. Mean number of cells positive for CB1 receptor per high power field expression of brain areas involved in emotional circuit. [file 40360_2023_644_MOESM1_ESM.docx]

|  | **Control ±SD** | **Low dose ±SD** | **High dose ±SD** |
| --- | --- | --- | --- |
| **Male** | 13.9 (±1.2) | 27.6 (±1.2)* | 36.7 (±1.7)*≠ |
| **Female** | 22.7 (±1.5) | 34.3 (±1.0)* | - 1. (± 2.2)*≠ |

**Table S1**: Mean number of cells positive for CB1 receptor per high power field expression of brain areas involved in emotional circuit.

One-way ANOVA test was carried out. *Significant compared to control groups; ≠ significant compared to the low dose group of the same gender & to the other gender of the same dose group. SD: standard deviation.
